# Obesity, Inflammation, and Tumor Microenvironment in Three-Dimensional Models of Breast Cancer

**DOI:** 10.3390/cells15090761

**Published:** 2026-04-24

**Authors:** Yarely M. Salinas-Vera, Yussel Pérez-Navarro, Jonathan Puente-Rivera, María Elizbeth Álvarez-Sánchez, César López-Camarillo

**Affiliations:** 1 Posgrado en Ciencias Genómicas, Universidad Autónoma de la Ciudad de México, Ciudad de México 03100, Mexico; yussel.perez@estudiante.uacm.edu.mx (Y.P.-N.); maria.alvarez@uacm.edu.mx (M.E.Á.-S.); 2 División de Investigación, Hospital Juárez de México, Ciudad de México 07760, Mexico; jonathan.puente@salud.gob.mx

**Keywords:** breast cancer, obesity, inflammation, tumor microenvironment, adipocytes, 3D culture, co-culture

## Abstract

**Highlights:**

**What are the main findings?**

- Obesity reprograms adipose tissue dynamics.
- This process involves systemic and local inflammation, metabolic changes, and hormonal alterations, which impact breast cancer development and progression.
- The review emphasizes the use of three-dimensional co-culture models, which more accurately replicate the breast tumor microenvironment, providing a better platform for studying adipocyte–tumor cell interactions.

**What are the implications of the main findings?**

- Targeting the obesity-induced changes in adipose tissue could provide new therapeutic opportunities for treating breast cancer associated with patient obesity.
- The use of 3D co-culture models of adipocytes/cancer cells highlights the need for advanced experimental platforms that better mimic the complexity of the tumor microenvironment.
- This could potentially lead to the discovery of novel targets for more effective and personalized treatments for obese breast cancer patients.

**Abstract:**

Obesity is recognized as a risk factor for breast cancer development and progression. Adipocytes exert their oncogenic effects through complex and interconnected biological mechanisms that encompass metabolic dysfunction, chronic low-grade inflammation, and systemic endocrine alterations. Herein, we reviewed the current evidence explaining how obesity induces a state that reprograms adipose tissue and remodels the breast cancer tumor microenvironment (TME). We first discuss the systemic and local mechanisms linking obesity to inflammation and how these alterations reshape the functional organization of the mammary gland. Then, we discuss how the chronic exposure to tumor-derived signals, together with the altered metabolic state of obese adipose tissue, induces a functional reprogramming of adipocytes, giving rise to so-called cancer-associated adipocytes (CAAs), which actively contribute to tumor progression. Also, the strengths and limitations of biological models to study the crosstalk between adipocytes and tumor cells, including two-dimensional (2D) monolayers and three-dimensional (3D) cell cultures, as well as animal models, are discussed. Special emphasis is placed on 3D co-culture models, which more accurately reproduce spatial organization, direct cell–cell contact, and diffusion dynamics, providing a more physiologically relevant environment for studying how obesity and inflammation reshape the TME in breast cancer. Finally, we highlight the limitations of conventional experimental models and review recent advances in 3D-based platforms, emphasizing their mechanistic insights and translational potential.

## 1. Introduction

Breast cancer (BC) remains the most frequently diagnosed malignancy in women and one of the leading causes of cancer-related mortality worldwide, representing a substantial public health and socioeconomic burden [1]. In parallel, obesity has reached epidemic proportions globally, and its coexistence with breast cancer has consistently been associated with increased disease incidence, more aggressive tumor progression, and poorer clinical outcomes, particularly in hormone-dependent subtypes [2,3]. Unlike many other risk factors, obesity exerts its oncogenic effects through complex and interconnected biological mechanisms that encompass metabolic dysfunction, chronic low-grade inflammation, and systemic endocrine alterations. This persistent inflammatory state, initially recognized as a hallmark of obesity, originates from the dysfunctional expansion of adipose tissue and is sustained by the continuous release of pro-inflammatory cytokines, adipokines, and metabolic intermediates [4,5]. These mediators not only disrupt systemic metabolic homeostasis but also directly influence the behavior of mammary epithelial cells and the tumor-associated stroma.

In BC, this interaction is particularly relevant due to the adipose-rich composition of the mammary gland. Mammary adipose tissue is not a passive structural component of the stroma but rather a dynamic endocrine and immunomodulatory organ capable of regulating local inflammation, tissue metabolism, and paracrine signaling. Clinical and experimental studies have demonstrated that, in obesity, mammary adipose tissue acquires a pro-inflammatory phenotype characterized by immune cell infiltration, crown-like structures (CLSs), and elevated levels of inflammatory mediators, which correlate with increased tumor aggressiveness and adverse clinical outcomes [2,6]. Concurrently, the concept of the tumor microenvironment (TME) has evolved toward an integrative framework that recognizes its active role in cancer initiation, progression, and therapeutic resistance. The obesity-associated breast cancer TME displays distinct features, including peri-adipocytic inflammation, metabolic reprogramming, extracellular matrix (ECM) remodeling, and immune modulation, all of which promote tumor adaptation to adverse metabolic conditions [7,8]. These processes are tightly interconnected and are further amplified by the chronic low-grade inflammatory milieu characteristic of the obese state. Despite substantial advances in the molecular characterization of these mechanisms, much of the available evidence derives from two-dimensional in vitro systems and animal models that fail to adequately recapitulate the spatial, metabolic, and inflammatory complexity of the human obesity-associated TME [9]. These limitations have driven the development of advanced three-dimensional experimental models, including spheroids, organotypic cultures, microfluidic systems, and bioengineered platforms, which enable a more physiologically relevant integration of tissue architecture and functional interactions among adipocytes, tumor cells, and stromal components [10,11].

In this review, we critically examine how obesity induces a chronic, low-grade inflammatory state that reprograms adipose tissue and remodels the breast cancer TME. We first discuss the systemic and local mechanisms linking obesity to inflammation, followed by an analysis of how these alterations shape the functional organization of the mammary TME. Finally, we highlight the limitations of conventional experimental models and review recent advances in three-dimensional platforms applied to obesity-associated breast cancer, emphasizing their mechanistic insights and translational potential.

## 2. Obesity and Low-Grade Chronic Inflammation

Obesity has emerged as an independent risk factor for the development, progression, and poorer prognosis of breast cancer, mainly due to its association with a state of chronic low-grade inflammation. Unlike acute inflammation, this condition is persistent, metabolically driven, and systemic and originates primarily from the dysfunctional expansion of adipose tissue. The interplay among adipose tissue dysfunction, sustained release of inflammatory mediators, and endocrine and metabolic alterations establishes a protumorigenic milieu that directly affects the mammary gland. The following subsections integrate these processes and highlight their mechanistic relevance to breast cancer biology.

### 2.1. Obesity-Associated Adipose Tissue Dysfunction

Pathological expansion of adipose tissue in obesity leads to a progressive loss of functional plasticity, transforming it into a metabolically dysfunctional and inflammatory organ. Excessive expansion of white adipose tissue occurs predominantly through adipocyte hypertrophy, resulting in local hypoxia, endoplasmic reticulum stress, mitochondrial dysfunction, and increased adipocyte death [12,13]. These events promote the release of free fatty acids, pro-inflammatory cytokines, and damage-associated molecular patterns (DAMPs), which act as potent inducers of chronic inflammation. Consequently, obese adipose tissue undergoes profound architectural remodeling, including fibrosis, ECM alterations, and marked infiltration of immune cells. Pro-inflammatory macrophages accumulate around damaged adipocytes, forming CLSs that constitute a major source of tumor necrosis factor alpha (TNF-α), interleukin-6 (IL-6), and interleukin-1 beta (IL-1β) [14,15]. In the mammary tissue, dysfunction of the peritumoral adipose compartment disrupts local homeostasis. It promotes activation of inflammatory and oncogenic programs in the mammary epithelium, thereby contributing to a microenvironment permissive for malignant transformation and tumor progression [2].

### 2.2. Systemic Inflammatory Mediators

In breast cancer, mediators such as IL-6 and TNF-α play central roles by activating key signaling pathways, including Nuclear Factor kappa B (NF-κB), Janus kinases (JAK)/Signal Transducers and Activators of Transcription (STAT), and mitogen-activated protein kinase (MAPK), which regulate cell proliferation, survival, angiogenesis, and epithelial–mesenchymal transition (EMT) [16,17]. Moreover, obesity-associated systemic inflammation may impair antitumor immune surveillance, fostering an immunologically permissive environment that has been linked to increased tumor aggressiveness, therapeutic resistance, and poorer clinical outcomes in breast cancer patients [8]. Inflammation is a common denominator of multiple comorbidities associated with obesity, including cancer, which suggests that obesity-induced inflammation represents a shared underlying pathogenic mechanism that contributes to various health risks associated with this condition [2,18]. Given the focus of this review, we concentrate on fat tissue inflammation and its mechanistic and functional links to the development and progression of breast cancer, emphasizing how systemic metabolic inflammation converges with local interactions among fat cells, tumor cells, and the stroma in the mammary microenvironment [4,19].

### 2.3. Endocrine and Metabolic Alterations

Chronic low-grade inflammation in obesity is tightly interconnected with profound endocrine and metabolic alterations that directly modulate tumor biology. A hallmark feature is dysregulated adipokine secretion, characterized by elevated leptin levels and a marked reduction in adiponectin. Leptin acts as a pro-inflammatory and pro-tumor genetic mediator, activating signaling pathways such as phosphatidylinositol 3-kinase (PI3K)/protein kinase B (AKT), JAK/STAT, and MAPK and promoting cell proliferation, angiogenesis, and tumor cell survival in breast cancer [20]. In contrast, adiponectin exerts anti-inflammatory and antiproliferative effects; thus, its reduction removes a key protective signal against tumor progression. Additionally, obesity is associated with insulin resistance and compensatory hyperinsulinemia, which increases the bioavailability of growth factors and potentiates mitogenic signaling in mammary cells [21]. Alterations in lipid metabolism and elevated circulating fatty acids further contribute to oxidative stress and tumor metabolic reprogramming, facilitating cancer cell adaptation to inflammatory and metabolically adverse environments. Collectively, these endocrine and metabolic disruptions act synergistically with chronic inflammation to establish a central pathophysiological axis linking obesity to breast cancer initiation and progression.

Additionally, in postmenopausal women, obesity increases the risk of breast cancer due to a combination of hormonal and inflammatory changes [22,23]. After the menopause, adipose tissue becomes the primary source of estrogen, thanks to aromatase, which converts androgens into estrone [24]. This process is particularly intense in obese women, as increased fat tissue enhances aromatase activity, leading to higher levels of estrone. Estrone, as a potent cellular activator, promotes the proliferation and survival of tumor cells, especially in estrogen receptor-positive (ER+) breast cancers. But it is not just about estrogen production: the pro-inflammatory state induced by obesity, with elevated levels of cytokines such as IL-6, TNF-α, and leptin, further amplifies tumor progression [25]. It does this by enhancing local estrogen signaling and promoting an inflammatory microenvironment that favors cancer growth [26]. All of this underscores the increased risk of breast cancer in obese postmenopausal women and highlights the importance of controlling obesity to reduce risk and improve outcomes in this population.

## 3. Tumor Microenvironment in Breast Cancer and Obesity

The TME of breast cancer constitutes a highly specialized biological niche, shaped mainly by the abundance of mammary adipose tissue and by the continuous interplay between tumor cells and stromal components. In the context of obesity, the systemic alterations described above are translated locally into a functional reprogramming of the TME, characterized by metabolic, structural, and immunoregulatory changes that collectively promote tumor progression.

### 3.1. Reprogramming of Mammary Adipocytes and Formation of Cancer-Associated Adipocytes in Obesity

**Figure 1:**
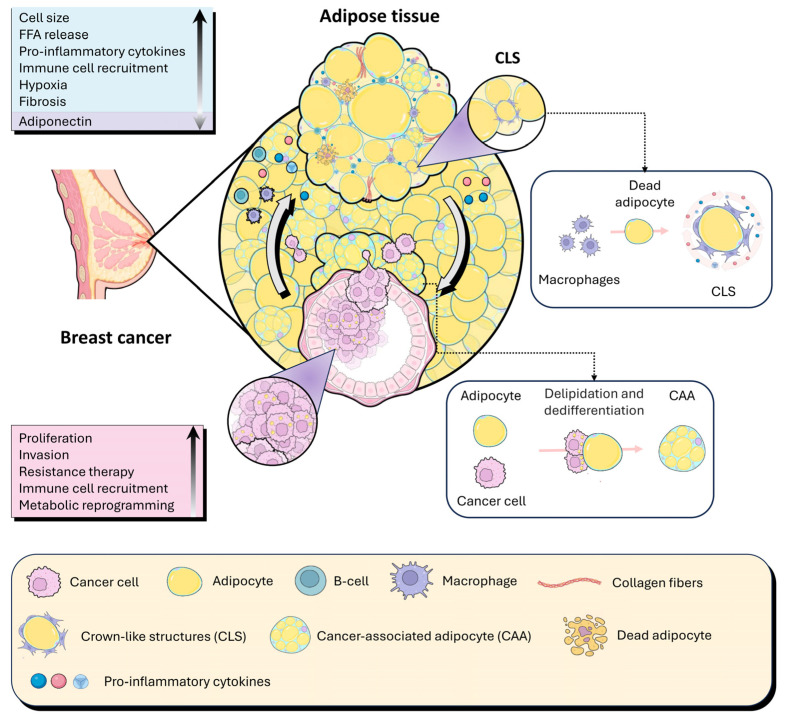
Interaction between adipose cells and breast cancer cells in the tumor microenvironment associated with obesity. In obesity, adipose tissue undergoes hypertrophy, which increases the secretion of pro-inflammatory cytokines. This inflammatory environment promotes the polarization and recruitment of macrophages to the TME, contributing to the development of CLS, in which macrophages surround dying adipocytes, evidencing a cycle of chronic inflammation. The inflammation, along with oxidative stress and secretory dysfunction of the adipocytes, increases the release of FFA, insulin, and IGF-1, promoting a pro-tumorigenic environment. In the tumor, breast cancer cells interact with adipocytes, reprogramming them to induce lipolysis and atrophy, which results in the transfer of lipids to tumor cells. This process drives the proliferation, migration, invasion, and metastasis of cancer cells. In turn, the increased release of lipids and inflammatory cytokines from CAA favors metabolic reprogramming of the TME and contributes to tumor growth and therapy resistance. The TME associated with obesity is characterized by the alteration of adipose tissue, which becomes an active source of inflammation and the factor that promotes tumor aggressiveness. These bidirectional interaction processes between breast cancer cells and adipocytes reinforce each other, creating a cycle that favors the progression of breast cancer and metastasis.

In obesity-associated breast cancer, mammary adipocytes transition from passive energy-storing cells into dynamic components of the TME. Chronic exposure to tumor-derived signals, together with the altered metabolic state of obese adipose tissue, induces profound functional reprogramming of adipocytes, giving rise to so-called cancer-associated adipocytes (CAAs), which actively contribute to tumor progression [27] (Figure 1). Experimental evidence from in vitro models has characterized this process as a sequential program of adipocyte dedifferentiation. Specifically, adipocytes derived from murine 3T3-L1 and 3T3-F442A cell lines, when co-cultured with breast cancer cells or exposed to tumor-conditioned media, exhibit a progressive reduction in cell size and lipid content, accompanied by a marked decrease in the expression of mature adipocyte markers such as adiponectin, resistin, and FABP4 [27,28]. These changes are associated with the suppression of key adipogenic transcriptional regulators, peroxisome proliferator-activated receptor gamma (PPARγ) and CCAAT/enhancer-binding protein alpha (C/EBPα), leading to characteristic morphological alterations, including small, dispersed lipid droplets and loss of the differentiated adipocyte phenotype [28,29].

Concomitantly, CAAs acquire features that partially resemble those of brown or beige adipocytes, including overexpression of uncoupling protein 1 (UCP1) and re-expression of genes associated with preadipocytes, which promote proliferative capacity [30]. In parallel, they develop an activated inflammatory and stromal phenotype, characterized by the secretion of chemokines, pro-inflammatory cytokines, and matrix-remodeling proteases, thereby facilitating tumor invasion and remodeling of the microenvironment [29,31]. In addition, CAAs undergo actin cytoskeleton reorganization, express markers typical of activated fibroblasts, including fibroblast activation protein (FAP), and adopt a fibroblast-like morphology [32,33]. Metabolically, this reprogramming involves activating catabolic pathways and releasing energy metabolites such as lactate, pyruvate, ketone bodies, and free fatty acids. These are partly produced through tumor signal-induced lipolysis, driven by the activation of hormone-sensitive lipase (HSL). However, the exact role of adipocyte lipolysis remains debated, as tumor cells may also actively participate in this process. [27,33]. The pathophysiological relevance of adipocyte reprogramming has been validated in vivo, where adipocytes at the invasive front of human breast tumors exhibit reduced size and molecular profiles consistent with CAAs, in contrast to adipocytes farther from the tumor. Within the tumor core, the scarcity of mature adipocytes and the abundance of fibroblast-like cells support the hypothesis that a fraction of the fibroblast pool within the TME originates from dedifferentiated adipocytes. This concept is further reinforced by lineage-tracing studies in animal models, which demonstrate adipocytes’ capacity to give rise to adipocyte-derived fibroblasts and to contribute to the desmoplastic reaction associated with breast cancer [34,35].

### 3.2. Spatial Organization of Inflammation Within the Mammary TME

The functional reprogramming of mammary adipocytes described above does not occur in isolation but is embedded within a spatially organized inflammatory architecture of the mammary TME. In particular, CAAs function as local hubs of inflammatory signaling, contributing to the formation of inflammatory microdomains that modulate tumor progression in obesity [14,18]. One of the most representative manifestations of this spatial organization is the formation of CLS in mammary adipose tissue, characterized by macrophages surrounding damaged or dedifferentiated adipocytes [14,36]. These structures are predominantly localized in peritumoral regions and are associated with high local production of pro-inflammatory cytokines, including IL-6, TNFα, and IL-1β, as well as sustained activation of oncogenic signaling pathways such as NF-κB and STAT3 in adjacent tumor cells. The presence of CLS in breast cancer has been correlated with obesity, higher tumor grade, and poorer clinical outcomes [36].

Spatial organization of inflammation also extends to the vascular compartment of the mammary TME. In regions enriched in CAAs and inflammatory mediators, activated endothelial cells exhibit increased expression of adhesion molecules like Intercellular Adhesion Molecule-1 (ICAM-1) and Vascular Cell Adhesion Molecule-1 (VCAM-1), thereby facilitating selective recruitment of immune cells to specific inflammatory niches within the tumor [18,37]. This process establishes local inflammatory gradients that favor protumorigenic polarization of tumor-associated macrophages (TAMs) and reinforce functional crosstalk between reprogrammed adipose tissue and cancer cells [37]. At the molecular level, tumors from patients with estrogen receptor-positive (ER+) breast cancer and obesity show significant enrichment of gene expression signatures associated with chronic inflammation and immune cell trafficking compared with tumors from non-obese patients, indicating persistent local inflammatory activation. These alterations suggest that adipokines and inflammatory mediators derived from adipose tissue actively contribute to ER+ tumor biology, beyond effects mediated solely by systemic estrogens, insulin, or IGF-1. Notably, these signals converge on the activation of key oncogenic pathways, such as PI3K/AKT/mTOR, supporting the notion that obesity promotes ER+ breast cancer progression through a functional axis that integrates local inflammation and adipose tissue-derived signaling [38,39].

Consistently, mammary tissue from obese women with breast cancer exhibits an exacerbated local inflammatory state, characterized by the presence of periadipocytic inflammatory structures and a significant increase in aromatase expression in mammary adipocytes. Local inflammatory signals induce this increase and enhance intratumoral estrogen production, establishing a direct functional link between adipose microenvironment inflammation, local hormonal signaling, and breast cancer progression [6]. Finally, advances in spatial transcriptomics have confirmed that the breast cancer TME displays a defined spatial organization, in which gene expression patterns of tumor, immune, and stromal compartments are distributed into functional regional domains. By preserving tissue architecture, these approaches capture the spatial heterogeneity of the TME and provide an integrative framework supporting the biological and clinical relevance of spatially organized inflammatory processes in the mammary TME [40].

### 3.3. Metabolic Coupling Between Adipocytes and Tumor Cells

In addition to producing inflammatory mediators, cancer-CAAs act as metabolic suppliers within the mammary TME. The strong interaction between reprogrammed adipocytes and tumor cells establishes a functional metabolic link that supports tumor metabolic and biosynthetic adaptations, particularly in obesity-associated cases. [18]. This coupling is characterized by bidirectional metabolite exchange and coordinated reprogramming of metabolic pathways in both cellular compartments [35].

One of the best-characterized mechanisms underlying this coupling is lipid transfer from adipocytes to tumor cells. Seminal studies demonstrated that breast cancer cells induce lipolysis in neighboring adipocytes, promoting the release of free fatty acids that are actively taken up by tumor cells via transporters such as CD36 and fatty acid-binding proteins (FABPs) [41,42]. These lipids are subsequently utilized as energy substrates through mitochondrial β-oxidation or incorporated into biosynthetic pathways, thereby supporting tumor growth, invasion, and resistance to metabolic stress [43]. Concomitantly, direct contact with tumor cells induces profound metabolic reprogramming in adipocytes, which acquire a catabolic phenotype characterized by lipid depletion, mitochondrial alterations, and increased secretion of energetic metabolites. This process has been described as a form of “metabolic dedifferentiation” of adipocytes, effectively converting peritumoral adipose tissue into a dynamic nutrient reservoir for the tumor [29]. In this context, CAAs release not only fatty acids but also lactate, ketone bodies, and amino acids, which can be reutilized by cancer cells to sustain tumor metabolic plasticity [44]. Collectively, these findings support a model of metabolic compartmentalization within the TME, in which stromal cells adopt a catabolic state driven by aerobic glycolysis, autophagy, and lipolysis. In contrast, tumor cells exploit these catabolites to maintain an anabolic metabolism reliant on oxidative phosphorylation and β-oxidation. This functional coupling has been conceptualized as a form of “parasitic cancer metabolism,” in which tumor cells hijack host tissue catabolism to promote their growth, survival, and, potentially, therapeutic resistance [45]. Adipocyte–tumor cell metabolic coupling is also tightly integrated with the inflammatory signaling described in the previous section. Cytokines such as IL-6 and TNFα, which are abundant in regions enriched in CAAs and CLS, potentiate adipocyte lipolysis and promote the expression of lipid metabolism-associated genes in tumor cells [2,18,46] (Figure 1). This convergence of inflammation and metabolism reinforces a positive feedback loop that further accelerates tumor progression, particularly in obesity-associated breast cancer. At the molecular level, multiple studies have shown that uptake of adipocyte-derived lipids activates key oncogenic pathways in tumor cells, including PI3K/AKT/mTOR signaling and peroxisome proliferator-activated receptors (PPARs), thereby promoting cell survival, migration, and resistance to systemic therapies [20,47,48]. In breast cancer, this metabolic coupling has been associated with more aggressive phenotypes, enhanced metastatic potential, and reduced responsiveness to treatment, underscoring its clinical relevance.

Taken together, metabolic coupling between reprogrammed adipocytes and tumor cells constitutes a critical functional axis of the mammary TME, in which inflammatory, hormonal, and metabolic signals converge to sustain tumor plasticity and disease progression. This perspective further reinforces the concept of adipose tissue as an active regulator of tumor biology and a potential therapeutic target, particularly relevant in the setting of obesity (Figure 1).

### 3.4. Extracellular Matrix Remodeling

Extracellular matrix (ECM) remodeling is a dynamic and multifactorial process that plays a central role in breast cancer progression by regulating both the biomechanical properties of the tissue and the integration of biochemical cues within the TME. In the normal mammary gland, the ECM preserves epithelial architecture and tissue homeostasis; however, during tumorigenesis, profound alterations in ECM composition, fibrillar organization, and stiffness occur, thereby facilitating local invasion, metastatic dissemination, and therapeutic resistance [49,50]. In obesity, expansion of mammary adipose tissue is accompanied by chronic low-grade inflammation, which further exacerbates aberrant remodeling of the peritumoral ECM. CAAs, together with cancer-associated fibroblasts (CAFs) and infiltrating immune cells, act as active regulators of this process. Breast cancer studies have shown that reprogrammed CAAs display increased expression of matrix metalloproteinases (MMPs), including MMP-2, MMP-9, and MMP-11, as well as cathepsins, thereby promoting degradation of collagen, laminin, and other ECM glycoproteins and facilitating tumor cell invasion into the surrounding stroma [29,51].

Concomitantly, persistent activation of CAFs drives excessive, disorganized deposition of ECM components, including type I collagen, fibronectin, and tenascin-C, thereby increasing stromal density and stiffness in mammary tissue. Directional alignment of collagen fibers, particularly at the invasive front, has been identified as a structural hallmark associated with enhanced migratory capacity and increased metastatic risk in breast tumors [52,53]. These mechanical alterations in the ECM activate mechano-transduction pathways in tumor cells, including integrin-mediated signaling, focal adhesion kinase (FAK), and the transcriptional co-activators YAP/TAZ, thereby promoting EMT, cell motility, and adaptation to adverse microenvironmental conditions [54,55]. Obesity-associated inflammation amplifies these processes through the sustained release of pro-inflammatory cytokines and profibrotic factors, such as IL-6, TNF-α, and TGF-β, particularly in regions enriched for CLS (Figure 1). These signals not only stimulate fibroblast activation and ECM remodeling but also establish positive feedback loops linking inflammation, matrix stiffening, and oncogenic signaling [36]. Consequently, the remodeled ECM functions as an active scaffold that integrates inflammatory, metabolic, and mechanical signals, thereby enhancing tumor aggressiveness and contributing to intratumoral heterogeneity in breast cancer [50]. Collectively, ECM remodeling in breast cancer is a dynamic process driven by the interplay among reprogrammed adipocytes, fibroblasts, and immune cells within the TME, and particularly exacerbated in the setting of obesity. This perspective positions the ECM not merely as a passive structural component, but as an active regulator of tumor progression and a potential therapeutic target for strategies aimed at disrupting mechanical and stromal signaling in breast cancer.

### 3.5. Local Hypoxia and Tumor Adaptation Induced by the Obesogenic Tumor Microenvironment

Hypoxia is a defining feature of the breast cancer TME and a powerful driver of tumor adaptation, malignant progression, and therapeutic resistance [56]. In the context of obesity, expansion of mammary adipose tissue, together with aberrant stromal remodeling and vascular dysfunction, promotes the development of persistent hypoxic regions within the TME [42]. These conditions impose intense selective pressures that favor activation of adaptive transcriptional programs in tumor cells, enabling survival and proliferation in environments with limited oxygen availability [57,58].

At the molecular level, the hypoxic response in breast cancer is primarily mediated by stabilization of Hypoxia-Inducible Factor 1-alpha (HIF-1α) and Hypoxia-Inducible Factor 2-alpha (HIF-2α), which regulate the expression of genes involved in angiogenesis, metabolism, invasion, and immune evasion [59]. In mammary tumors, HIF-1α activation has been associated with increased expression of Vascular Endothelial Growth Factor (VEGF), Glucose Transporter 1 (GLUT1), Carbonic Anhydrase 9 (CA9), and glycolytic enzymes, thereby promoting angiogenesis, vasculogenic mimicry, metabolic reprogramming toward aerobic glycolysis, and acidification of the TME [59,60,61]. Obesity further amplifies these responses by fostering a chronic inflammatory state characterized by sustained cytokine release, including IL-6 and TNFα, which can stabilize HIF-1α in a hypoxia-independent manner and induce hypoxic signaling within the mammary TME [62,63].

Local hypoxia also closely intersects with ECM remodeling and vascular dysfunction, hallmark features of the obesogenic TME [64]. Increased stromal stiffness, mechanical compression of blood vessels, vasculogenic mimicry, and aberrant angiogenesis collectively contribute to inefficient perfusion and the generation of heterogeneous oxygen gradients within tumors [8]. In this setting, hypoxic regions are frequently associated with the invasive front and with dysfunctional perivascular niches, thereby favoring EMT, collective invasion, and metastatic dissemination in breast cancer [55,65]. Moreover, hypoxia induced by the obesogenic TME profoundly modulates the composition and function of the tumor immune compartment [60,64]. In breast cancer, HIF-dependent signaling promotes macrophage polarization toward a protumorigenic phenotype, the exclusion of cytotoxic T lymphocytes, and the upregulation of immunosuppressive molecules, collectively contributing to immune evasion. These alterations are further reinforced by the interplay among hypoxia, inflammation, and adipokines, consolidating a microenvironment that is highly permissive for tumor progression and resistance to targeted therapies and immunotherapy [57,58].

From a translational perspective, numerous studies have demonstrated that tumor hypoxia in breast cancer is associated with poorer clinical outcomes, increased tumor aggressiveness, and resistance to chemotherapy, radiotherapy, and targeted therapies [57]. In this regard, three-dimensional models that incorporate adipocytes, oxygen gradients, and stromal architecture have emerged as relevant platforms to investigate tumor adaptation to hypoxia in an obesogenic context, as they more faithfully recapitulate the spatial and functional heterogeneity of the mammary TME. Collectively, obesity-induced local hypoxia should be considered a key integrative axis linking inflammation, metabolic reprogramming, stromal remodeling, and immune evasion in breast cancer and represents a promising target for the development of combined therapeutic strategies.

## 4. Limitations of Conventional Models

Despite the growing body of evidence positioning the obesogenic TME as a key regulator of breast cancer progression, much of the available mechanistic insight has been derived from conventional experimental models that only partially capture the biological complexity of these interactions [66]. Two-dimensional (2D) culture systems and animal models have been instrumental in identifying central oncogenic pathways; however, they present inherent limitations that constrain their ability to faithfully recapitulate tissue architecture, metabolic heterogeneity, and the spatial organization that define the mammary TME in the context of obesity [67] (Table 1).

### 4.1. Limitations of Two-Dimensional (2D) Models

**Figure 2:**
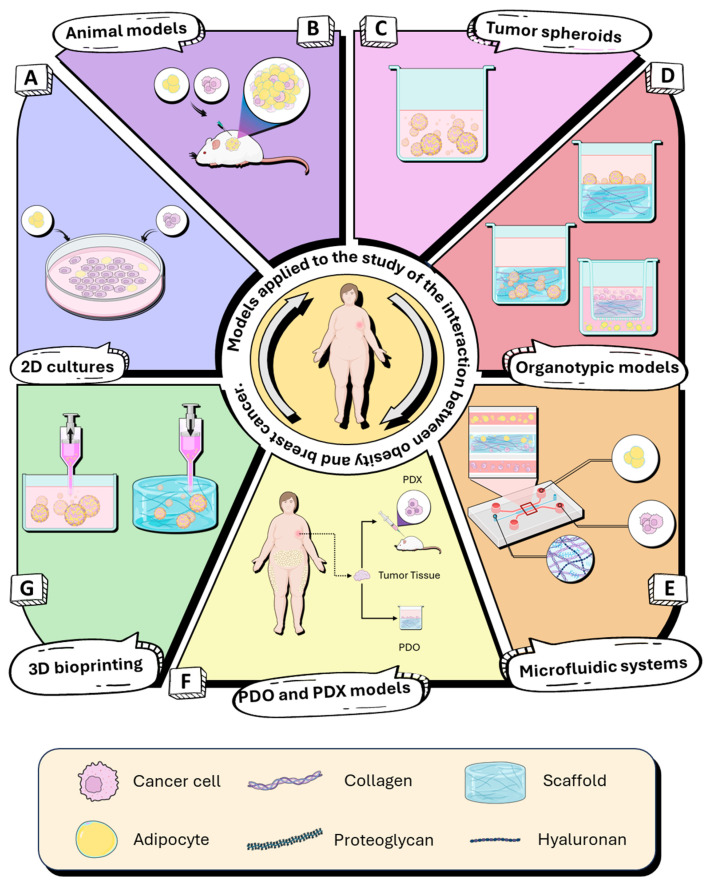
Models applied to the study of the interaction between obesity and breast cancer. (**A**) Two-dimensional (2D) in vitro cultures of breast cancer cells and adipocytes. (**B**) In vivo breast cancer models incorporating adipose tissue. (**C**) Three-dimensional (3D) tumor spheroid co-cultures of breast cancer cells and adipocytes under low-adhesion or suspension conditions. (**D**) Three-dimensional organotypic co-culture models based on extracellular matrices or Transwell systems. (**E**) Compartmentalized microfluidic co-culture systems under continuous flow. (**F**) PDO and PDX models, showcasing patient-derived organoids (PDOs) and patient-derived xenografts (PDX) to replicate tumor heterogeneity and therapeutic responses in obesity-associated breast cancer. (**G**) Three-dimensional bioprinting models with spatially controlled deposition of breast cancer cells and adipocytes. Key: Breast cancer cell; adipocyte; collagen; proteoglycan; hyaluronan; scaffold.

Monolayer cell cultures remain widely used because of their technical simplicity and experimental reproducibility. However, these systems lack many critical components of the mammary TME (Figure 2A) [11,67]. Under 2D conditions, tumor cells are exposed to a homogeneous availability of oxygen and nutrients and are deprived of three-dimensional interactions with the ECM, adipocytes, and other stromal cell types [66]. As a consequence, key processes such as localized inflammation, formation of metabolic gradients, regional hypoxia, and adipocyte–tumor cell metabolic coupling cannot be modeled in a physiologically relevant manner [68].

This limitation is particularly evident in studies of adipose tissue. Mature adipocytes rapidly lose their differentiated phenotype or undergo apoptosis in 2D culture, precluding sustained evaluation of their interactions with breast cancer cells [69]. Consequently, central features of the obesogenic TME, such as tumor-induced lipolysis, adipokine secretion, and the transfer of energetic metabolites, are underrepresented or absent [29]. In addition, therapeutic response studies performed in 2D cultures tend to overestimate the efficacy of chemotherapeutic and targeted agents, as they fail to account for stromal-mediated protection, ECM stiffness, and hypoxic niches, all of which are closely associated with therapy resistance in patients with obesity [64].

### 4.2. Strengths and Limitations of Animal Models

Animal models, particularly genetically engineered mouse models and diet-induced obesity models, have been instrumental in establishing causal links between obesity, chronic inflammation, increased aromatase activity, and breast cancer progression (Figure 2B) [70,71]. These systems have been critical for demonstrating the contribution of estrogen-driven inflammatory signaling in ER-positive tumors and the systemic impact of energy metabolism on tumor growth [14,19]. Nevertheless, extrapolating these findings to human disease poses substantial challenges. Significant differences exist between mice and humans in mammary tissue organization, adipose tissue distribution, immune composition, and adipocyte biology. Notably, murine adipocytes differ in size, lipolytic capacity, and inflammatory profile, which can markedly influence metabolic coupling between the adipose stroma and tumor cells [14].

Furthermore, many animal models do not adequately recapitulate the intratumoral heterogeneity or the spatial organization of the TME observed in human tumors [72]. Accelerated tumor progression and the lack of prolonged exposure to an obesogenic inflammatory state limit the ability to model long-term adaptive processes, such as metabolic compartmentalization, progressive ECM remodeling, and the evolution of therapeutic resistance [73].

### 4.3. Translational Gaps and the Need for Advanced Experimental Systems

Taken together, the limitations of 2D and animal models contribute to a particularly relevant translational gap in the study of obesity-associated breast cancer. Mechanisms identified in reductionist systems do not always predict tumor behavior in contexts where stromal complexity, metabolic plasticity, and spatial organization are critical determinants [74]. This disconnect is reflected in the limited performance of therapies targeting inflammatory or metabolic pathways that show promising results in preclinical settings but only modest benefits in the clinic [75]. Moreover, conventional models rarely incorporate key clinical variables such as body mass index, adipose tissue distribution, or menopausal status, all of which decisively influence breast cancer biology. These limitations underscore the urgent need for advanced experimental platforms that preserve three-dimensional architecture, integrate multiple cellular compartments, and recapitulate the metabolic and mechanical gradients characteristic of the obesogenic TME [38].

In this context, three-dimensional and organotypic models are emerging as essential tools that can excite researchers about new avenues for connecting mechanistic insights with clinical outcomes, laying a foundation for the innovative approaches discussed in the following sections.

## 5. Three-Dimensional Models Applied to the Study of Obesity and Inflammation

Mammary adipose tissue is a critical component of the TME in breast cancer, particularly in obesity, where adipocytes acquire a metabolically active, pro-inflammatory phenotype. Unlike two-dimensional cultures, 3D co-culture models more accurately reproduce spatial organization, direct cell–cell contact, and diffusion dynamics, thereby providing a more physiologically relevant environment for studying how obesity and inflammation reshape the TME in breast cancer (Table 1).

### 5.1. Tumor Spheroids Co-Cultured with Adipocytes

Three-dimensional spheroid-based co-culture models with adipocytes have become fundamental tools for investigating the functional interactions between breast cancer cells and adipocytes within the tumor microenvironment, particularly in the context of obesity and chronic inflammation [76]. Multicellular tumor spheroids are among the most widely used three-dimensional (3D) models for studying cell–cell communication between adipocytes and breast cancer cells [77]. As shown in Figure 2C, tumor cells co-cultured with mature adipocytes self-organize into compact three-dimensional tumor spheroids that reproduce gradients of oxygen, nutrients, and metabolites similar to those observed in vivo. These tumor spheroids are generated via seeding in ultra-low-attachment plates or in spinner flasks, which prevent substrate adhesion and promote three-dimensional organization [11,78]. This three-dimensional organization promotes physiologically relevant cell–cell interactions that are not observed in conventional two-dimensional cultures. Numerous studies have demonstrated that co-culture of breast cancer tumor spheroids with adipocytes enhances tumor cell proliferation, invasive capacity, and metabolic reprogramming, supporting the view of adipose tissue as an active functional component of the TME rather than merely an energy reservoir [79,80]. Notably, tumor spheroids derived from breast cancer cell lines such as MDA-MB-231 or MCF-7, when cultured in three-dimensional co-culture with differentiated adipocytes or adipose-derived stromal cells, consistently exhibit increased tumor cell migration and invasion, associated with activation of inflammatory signaling pathways. In particular, robust activation of the C-C Motif Chemokine Ligand 5/C-C Motif Chemokine Receptor 1 (CCL5/CCR1) chemokine axis has been described in 3D systems, resulting in a significant increase in cellular motility compared with two-dimensional co-cultures, underscoring the critical dependence of these signaling events on the three-dimensional context [81].

Among the spheroidal 3D models used in this context, it is essential to distinguish between conventional tumor spheroids and mammospheres [82,83]. While tumor spheroids are aggregates of heterogeneous tumor cell populations and are primarily used to study tumor architecture, invasion, and stromal interactions, mammospheres are three-dimensional structures enriched for subpopulations with cancer stem cell-like properties. Thus, they are used to investigate self-renewal and cellular plasticity [84]. Despite these methodological and conceptual differences, both systems share a three-dimensional organization that enables the analysis of functional interactions with adipocytes in a spatially relevant context. In this regard, studies using mammospheres have complemented findings from conventional tumor spheroids by revealing specific mechanisms of metabolic communication between breast cancer cells and adipose tissue. Recurrent evidence indicates that breast cancer mammospheres secrete factors that induce lipolysis and browning programs in adjacent adipocytes, thereby increasing the availability of energy substrates for tumor cells. Collectively, the literature suggests that both tumor spheroids and mammospheres, when co-cultured with adipocytes, provide complementary perspectives on the bidirectional nature of adipocyte–tumor communication, thereby consolidating spheroid-based 3D models as key tools for investigating the obesity-associated TME in breast cancer [85].

### 5.2. Organotypic Cultures of Tumor Cells in Co-Culture with Adipocytes

Three-dimensional organotypic co-culture models have been established as fundamental tools for investigating the functional interplay between breast cancer cells and adipose tissue, as they enable the reconstruction of tissue architecture, stromal biomechanical properties, and the spatial proximity between the two cellular compartments [81,84]. Compared with spheroid-based systems, these models incorporate complex extracellular matrices, such as type I collagen, Matrigel, or synthetic hydrogels, which facilitate cell–cell and cell–ECM interactions that more closely resemble the in vivo TME [11,84]. Figure 2D illustrates the different organotypic culture systems used to study the interaction between breast cancer cells and adipocytes. These models include three-dimensional heterotypic cultures, in which both cell types are embedded in extracellular matrices or cultured in on-top systems, allowing direct cell–cell and cell–ECM interactions. Additionally, organotypic models based on Transwell systems have been developed, enabling the study of paracrine communication between tumor cells and adipocytes under spatially controlled conditions, while maintaining physical separation between cellular compartments. Together, these approaches more accurately recapitulate the structural, metabolic, and inflammatory complexity of the tumor microenvironment associated with obesity [80,82].

Early three-dimensional organotypic studies, although not explicitly focused on cancer, laid the conceptual foundations for understanding the relevance of epithelial–adipose communication in mammary tissue. In this context, Huss and Kratz (2001) demonstrated that the three-dimensional co-culture of mammary epithelial cells with adipocytes within a 3D matrix enables tissue self-organization and preserves key morphological features of human mammary tissue, establishing one of the earliest bioengineered mammary tissue models [86]. Complementarily, Tong and coworkers showed that adipose tissue-derived mesenchymal stem cells acquire acinar-like structures when stimulated by mammary epithelial cells in three-dimensional culture, highlighting the instructive role of epithelial-derived signals on the adipose stroma [87].

Building on these observations, further research using three-dimensional organotypic breast cancer models has uncovered more complex influences of adipose tissue on tumor cell phenotypic plasticity. Notably, adipocytes and adipocyte-derived conditioned media partially induced a mesenchymal–epithelial transition (MET) in breast cancer cells with a mesenchymal phenotype, such as MDA-MB-231 and Hs578T, thereby promoting the formation of epithelial-like structures compared with the stellate colonies typically observed in control 3D cultures. In contrast, cell lines with a basal epithelial phenotype, such as SUM159 and MCF-7, exhibit less pronounced morphological changes. At the molecular level, adipocytes induce an incomplete MET without significantly affecting cell proliferation, while concomitantly, a reduction in lipid droplet size is observed in adipocytes under co-culture conditions, underscoring bidirectional metabolic communication [88]. Complementarily, organotypic platforms based on engineered anisotropic collagen scaffolds invested with adipocytes have been developed to investigate the combined influence of stromal architecture and adipose tissue on tumor behavior. These models demonstrated that adipocyte integration within anisotropically organized collagen matrices promotes directional tumor cell invasion and alters the response to chemotherapeutic agents, highlighting the regulatory role of stromal biomechanics in breast cancer aggressiveness [89].

The integration of omics-based approaches into three-dimensional organotypic models has enabled a more comprehensive understanding of adipose tissue-driven tumor reprogramming. Proteomic analyses in 3D co-culture systems have revealed coordinated alterations in the expression of proteins involved in lipid transport, hypoxia responses, and cytoskeletal organization, indicating that adipocyte–tumor interactions promote a systemic tumor adaptation to the adipose-rich microenvironment [90]. Finally, three-dimensional organotypic models have been adapted with a clear translational focus. In this context, highly complex multicellular systems integrating adipocytes, tumor cells, myoepithelial cells, macrophages, and fibroblasts within collagen matrices have been developed, enabling a more faithful recapitulation of the structural, inflammatory, and metabolic features of the obesity-associated tumor microenvironment. These models recapitulate the infiltration of tumor cells and macrophages into inflamed adipose tissue, a hallmark of breast cancer in obese patients, and have been used as preclinical platforms to evaluate therapeutic responses [91]. Collectively, these advances consolidate three-dimensional organotypic models as key tools for linking adipose tissue-induced molecular reprogramming with functionally and translationally relevant phenomena in breast cancer.

### 5.3. Three-Dimensional Adipocyte Cultures in Interaction with Breast Cancer Cells

Three-dimensional (3D) cultures of adipocytes in direct interaction with breast cancer cells have been developed as defined experimental models to systematically evaluate the functional effects of adipocytes on tumor phenotypes. In contrast to complex organotypic models or tumor spheroid-based systems, these approaches specifically focus on the adipocytic compartment and its bidirectional communication with cancer cells within a simplified three-dimensional environment [86,92].

Experimentally, these systems are established using adipocytes differentiated from human preadipocytes, cell lines such as 3T3-L1, or primary adipocytes, which are embedded in relatively simple three-dimensional matrices, including type I collagen, Matrigel, or synthetic hydrogels, and co-cultured either directly or in a compartmentalized manner with breast cancer cells [93]. From a molecular and functional perspective, adipocytes cultured in 3D retain an adipocytic identity that more closely resembles the in vivo state than that of cells cultured in a monolayer. Previous studies have shown that adipogenic differentiation in three-dimensional systems induces coordinated regulation of key genes associated with adipogenesis and lipid metabolism, including PPARγ, C/EBPα, FABP4, A-diponectin (ADIPOQ), Lipoprotein lipase (LPL), and Perilipin 1 (PLIN1), as well as components of the mitochondrial machinery and β-oxidation pathways. These 3D adipocytes also exhibit organized lipid accumulation and functional lipolytic responses to hormonal stimuli, confirming the preservation of metabolic function [94]. Complementarily, human adipocytes cultured in 3D have been shown to robustly recapitulate molecular programs associated with adipose tissue inflammation. Notably, the study by Soták M and coworkers demonstrated that 3D adipocytes exhibit a significant induction of pro-inflammatory mediators, including IL-6, IL-8, TNF-α, CCL2, and Prostaglandin–Endoperoxide Synthase 2 (PTGS2), as well as activation of NF-κB-related inflammatory pathways. Importantly, treatment with lipoxins consistently reduced the expression of these mediators at both the transcriptional and protein levels, validating 3D adipocytes as a metabolically and molecularly competent experimental system for the study of inflammatory processes [95]. These findings provide a robust molecular rationale for the use of 3D adipocytes in tumor-interaction studies.

When 3D-cultured adipocytes are co-cultured with breast cancer cells, these systems enable controlled analysis of adipocyte–tumor metabolic crosstalk under conditions that model leanness and obesity. Within this context, adipocytes maintain their identity, viability, and metabolic functionality in a three-dimensional environment, allowing direct comparison of the impact of adipose tissue metabolic status on tumor phenotype. Consistently, adipocytes derived from obese conditions exhibit an exacerbated inflammatory and metabolic profile, which is associated with increased tumor cell proliferation and modulation of responses to antineoplastic therapies. At the molecular level, co-culture induces activation of lipid metabolism, inflammation, and cell survival-related programs in tumor cells, consolidating these models as relevant experimental platforms for investigating obesity in breast cancer [69]. Overall, three-dimensional adipocyte cultures co-cultured with breast cancer cells constitute controlled experimental models that enable the dissection of adipocyte metabolic and inflammatory roles in tumor progression. Their ability to reproduce adipose tissue state-dependent differences positions them as highly relevant platforms for mechanistic studies of obesity-associated breast cancer.

### 5.4. Microfluidic “On-a-Chip” Systems

Microfluidic “on-a-chip” systems integrating adipose compartments with breast cancer cells have emerged as high-fidelity platforms for investigating TME dynamics under continuous flow and physiologically relevant gradients. Unlike static 3D models, these devices enable precise control of the physical and chemical microenvironment, allowing simulation of gradients of oxygen, nutrients, metabolic factors, and paracrine secretions that critically influence adipocyte–tumor communication [96]. In this context, Figure 2E illustrates a representative diagram of these systems. A pioneering example of such platforms is the generation of a human adipose microtissue within a microfluidic device, in which human adipose tissue-derived stem cells (ADSCs) differentiated in 3D to form a mass of functional adipocytes. These adipocytes expressed canonical adipose tissue markers and exhibited flow-modulated free fatty acid secretion, highlighting the importance of mechanical cues in regulating adipocyte function in on-chip systems [97]. Furthermore, tumor–adipose interaction studies using on-a-chip platforms have demonstrated that lipids released by 3D adipocytes activate HIF-1α in MDA-MB-231 breast cancer cells, thereby promoting the expression of genes associated with migration, invasion, and ECM degradation, including Ras-related C3 botulinum toxin substrate 1 (RAC1), Cell Division Control Protein 42 Homolog (CDC42), MMP2, and MMP9. This adipocyte–tumor metabolic crosstalk increased tumor cell motility, directional persistence, and invasive capacity. In parallel, adipokine and free fatty acid secretion reprogrammed tumor cell metabolism by enhancing fatty acid oxidation and mitochondrial activity, providing evidence that the adipose microenvironment acts as an active modulator of invasion within a controlled three-dimensional context. Collectively, these findings offer mechanistic insight into how adipocyte-derived metabolites function as oncometabolites in pro-metastatic processes [98]. Overall, on-a-chip systems enable controlled interrogation of how adipocytes modulate tumor migration, invasion, and metabolic plasticity through inflammatory and matrix-remodeling pathways, consolidating these platforms as physiologically relevant and valuable tools for mechanistic studies and therapeutic testing.

### 5.5. Patient-Derived Organoids (PDOs) and Patient-Derived Xenograft (PDX) Models

Patient-derived organoids (PDOs) and patient-derived xenografts (PDXs) are becoming established as key models for breast cancer research, as they more closely replicate tumor heterogeneity and the TME than traditional 2D cell cultures. These models offer significant advantages for studying tumor progression and treatment response, providing a more physiologically relevant system. PDOs are 3D cultures derived from patient tumor tissue that retain the key characteristics of the original tumor, including its architecture and genetic profile [99] (Figure 2F). These organoids provide a representative platform for studying tumor progression, invasion, and drug response while preserving the heterogeneity and architecture of the primary tumor [100]. Although PDOs have proven to be useful in breast cancer research, they are currently mainly focused on studies of drug response. So far, there are no reports addressing organoid models that analyze the inflammatory microenvironment associated with obesity in this disease, remaining as a knowledge gap.

On the other hand, PDX models, in which human tumor tissue is implanted into immunocompromised mice, preserve the tumor architecture and the TME, including adipocytes and stromal components [101] (Figure 2F). A recent study provided important information about the role of obesity-altered adipose stem cells (obASCs) in breast cancer metastasis, particularly in triple-negative breast cancer TNBC. The results of this study showed that obASCs secrete leptin, which activates a pro-metastatic phenotype in tumor cells, promoting migration and invasion through EMT. This study highlights the impact of adipocytes on tumor aggressiveness in obesity-associated breast cancer and how PDX models can be used to study these interactions [102].

### 5.6. Three-Dimensional Bioprinting of Adipocyte–Breast Cancer Co-Cultures

Three-dimensional (3D) bioprinting has emerged as a next-generation platform for modeling interactions between adipocytes and breast cancer cells, with unprecedented spatial and compositional control over cellular organization and the extracellular matrix [98]. Unlike other three-dimensional models, these technologies enable reproducible arrangement of multiple cell types and bioactive biomaterials, thereby facilitating the recreation of tissue heterogeneity, the biomechanical properties of the stroma, and the cell-proximity characteristics of the tumor microenvironment [103,104]. In the context of obesity, bioprinting models enable the integration of functional adipocytes with tumor cells, providing a highly biomimetic platform for studying metabolic, mechanical, and paracrine interactions. Figure 2G schematizes the application of 3D bioprinting models to investigate adipocyte–breast cancer interactions.

Unlike conventional 3D cultures, bioprinting enables the programmable organization of tumor cells, adipose tissue-derived stromal cells (ASCs), differentiated adipocytes, and biomimetic extracellular matrices, allowing reconstruction of tumor–adipose interfaces that are difficult to reproduce in static or microfluidic systems [103,104]. Pioneering studies using laser direct-write (LDW) approaches have demonstrated that fabricating biomimetic constructs with controlled spatial organization enables direct modeling of breast cancer cell invasion into adipose tissue. In these systems, the precise arrangement of adipocytes and tumor cells within extracellular matrices revealed that tumor invasion depends on adipose proximity and the three-dimensional microenvironmental architecture, providing one of the first experimental frameworks for studying tumor infiltration into adipose tissue in 3D [105].

Experimental evidence indicates that 3D-differentiated ASC spheroids maintain a functional adipocytic identity, as evidenced by sustained induction of PPARγ and FABP4. Co-culture with MDA-MB-231 cells triggered bidirectional molecular reprogramming, characterized within the adipose compartment by reduced lipid content and ECM remodeling, with increased expression of Collagen Type I Alpha 1 Chain (COL1A1), Collagen Type IV Alpha 1 Chain (COL6A1), and Fibronectin 1 (FN1), while tumor cells activated programs associated with migration and metabolic adaptation [106]. From a functional perspective, bioprinted adipose compartments directly influence tumor behavior. In spatially defined 3D bioprinted constructs, migration and invasion of MDA-MB-231 breast cancer cells were shown to depend critically on the proximity of adipose or ASC spheroids. Tumor cells exhibited significant increases in migration speed, directional persistence, and invasion distance, underscoring the instructive role of adipose tissue in activating invasive programs within a three-dimensional context [43]. Beyond direct adipocyte–tumor interactions, multicellular 3D bioprinted tumor models have demonstrated the capacity to recapitulate complex TME properties, including cellular heterogeneity, ECM remodeling, and resistance to immunotherapy. Notably, a 3D bioprinted tumor model reproduced immune exclusion states and microenvironment-driven differential therapeutic responses, validating these platforms as functional systems for studying stroma-dependent resistance mechanisms [104].

Collectively, three-dimensional bioprinting represents a modular, highly reproducible strategy for investigating adipose tissue-derived metabolic, inflammatory, and mechanical signals that regulate tumor progression. By enabling precise control over tissue architecture and cellular complexity, adipocyte–tumor bioprinted models provide a high-value conceptual and experimental framework for elucidating the mechanisms linking obesity, the tumor microenvironment, and therapeutic resistance in breast cancer.

**Table 1:** Comparison of experimental techniques for studying the interaction between obesity and breast cancer.

Model	Advantages	Limitations	Applications	References
2D Co-culture	Allows direct study of cell–adipocyte communication. Additionally, it is low-cost and easy to handle.	Lacks 3D complexity and extracellular matrix (ECM) and does not replicate metabolic and spatial gradients.	Study of crosstalk between adipocytes and tumor cells, metabolic changes, and inflammatory pathways	[10,67]
Animal Models (Diet-Induced Obesity)	Replicate the metabolic and systemic context, including tumor interaction with the obesogenic environment.	Species differences, ethical concerns, and difficulty replicating the TME.	Metastasis, systemic obesity and inflammation, estrogen signaling studies	[70,72]
3D Tumor spheroids	Better physiological representation allows gradients of oxygen and nutrients closer to in vivo conditions.	Lacks a scaffold that mimics the extracellular matrix (ECM) and size variability.	Tumor progression, metabolic coupling, inflammatory signaling, and invasion studies	[77,78]
3D Organotypic Cultures	Replicates tissue architecture and ECM–cell interactions.	Expensive, technically challenging, and requires complex materials such as Matrigel or hydrogels.	Study of tumor-adipocyte interaction, adipocyte differentiation, and ECM remodeling	[10,82]
Microfluidic “On-a-Chip” Systems	High precision in gradient control; realistic simulation of adipocyte–tumor communication.	Technical complexity, costly equipment, and scalability limitations.	Modeling metabolic and inflammatory crosstalk, invasion, migration, and therapeutic testing	[96]
PDOs and PDX	High clinical relevance, tumor heterogeneity is maintained, and reflects in vivo behavior.	Expensive, time-consuming, and limited tissue availability.	Potential for studying adipocyte–stroma–tumor interactions, metabolic reprogramming, and therapeutic responses.	[99,101]
3D Bioprinting Models	Spatial precision in cell organization allows recreation of the tumor and adipose microenvironment.	Requires advanced equipment, a complex setup, and a high cost.	Adipocyte–tumor interaction, ECM remodeling, metabolic reprogramming, and therapeutic response testing	[104]

## 6. Conclusions and Limitations

The evidence presented throughout this review consistently supports the role of obesity as a central modulator of breast cancer biology, acting primarily through a state of chronic low-grade inflammation that integrates metabolic dysfunction, endocrine alterations, and remodeling of the TME [5]. This persistent inflammatory context, originating in dysfunctional adipose tissue, operates at both systemic and local levels, promoting oncogenic signaling that favors tumor proliferation, cellular plasticity, immune modulation, and resistance to conventional therapies [4]. Within the mammary gland, the high proportion of adipose tissue confers relevance to these interactions. Under obese conditions, mammary adipose tissue becomes an active component of the tumor microenvironment, characterized by periadipocytic inflammation, sustained release of cytokines and adipokines, and metabolic reprogramming. These processes contribute to a pro-tumorigenic milieu and are associated with increased tumor aggressiveness and poorer clinical outcomes [2,38]. Importantly, these alterations exert differential effects depending on the molecular subtype of breast cancer, underscoring the need for stratified approaches that consider the patient’s metabolic status.

Despite significant advances in the molecular characterization of the obesity/inflammation/breast cancer axis, substantial limitations remain in the experimental models that underpin much of the current knowledge [8]. Two-dimensional systems oversimplify the spatial, metabolic, and inflammatory complexity of the tumor microenvironment, whereas animal models exhibit relevant physiological discrepancies in metabolic and immune regulation, thereby limiting direct extrapolation of findings to human disease [7,74]. In this context, three-dimensional models have emerged as key tools for capturing critical aspects of tissue architecture, intercellular communication, and metabolic gradients characteristic of the obesity-associated TME [9]. Nevertheless, these systems still face challenges related to standardization, reproducibility, and the simultaneous incorporation of multiple cellular compartments and systemic signals intrinsic to chronic low-grade inflammation [10,74]. Moreover, many current models lack a temporal dimension that would allow for the gradual progression of obesity-associated inflammation to be recapitulated.

It is important to highlight that therapeutic approaches targeting lipid metabolism, particularly fatty acids and fatty acid synthase (FASN), are emerging as potential strategies for treating obesity-associated breast cancer [107,108]. The inhibition of lipid synthesis and the modulation of lipid signaling pathways have shown promising results in preclinical studies, offering new avenues to address chemoresistance and tumor progression in obesity-related breast cancer [109]. PDO and PDX models can play a critical role in evaluating these lipid-targeted therapies, providing a more translational approach to developing personalized treatments for patients with obesity. In this regard, several promising studies have shown that FASN inhibitors, which block fatty acid synthesis, can reduce tumor growth and metastasis in preclinical models of breast cancer. These inhibitors effectively alter fatty acid metabolism in tumor cells that rely on lipids for growth and survival, particularly in obesity-associated breast cancer [109,110]. Additionally, inhibition of fatty acid synthesis sensitizes tumor cells to chemotherapy, thereby improving treatment response in preclinical models of resistant breast cancer [111].

Adipocytes in the TME also play a key role in the tumor aggressiveness of obesity-associated breast cancer. Adipocytes can secrete adipokines such as leptin, which promote metastasis and tumor invasion [75]. Targeting lipid pathways dependent on adipocytes is an emerging strategy to overcome chemoresistance and improve treatment outcomes in patients with obesity-related breast cancer.

## Data Availability

No new data were created or analyzed in this study.

## Abbreviations

The following abbreviations are used in this manuscript:

2D	Two-Dimensional
3D	Three-Dimensional
ADIPOQ	Adiponectin
ADSC	Adipose-Derived Stem Cell
ASC	Adipose Tissue-Derived Stromal Cell
ATMs	Adipose Tissue Macrophages
BC	Breast Cancer
BCCs	Breast Cancer Cells
CAA	Cancer-Associated Adipocyte
CA9	Carbonic Anhydrase 9
CDC42	Cell Division Control Protein 42 Homolog
C/EBPα	CCAAT/enhancer-binding protein alpha
CCL2	C-C Motif Chemokine Ligand 2
CCL5	C-C Motif Chemokine Ligand 5
CCR1	C-C Motif Chemokine Receptor 1
CLS	Crown-Like Structures
COL1A1	Collagen Type I Alpha 1 Chain
COL6A1	Collagen Type IV Alpha 1 Chain
DAMPs	Damage-Associated Molecular Patterns
ECM	Extracellular Matrix
EMT	Epithelial–Mesenchymal Transition
FABPs	Fatty Acid-Binding Proteins
FAK	Focal Adhesion Kinase
FFA	Free Fatty Acids
FASN	Fatty Acid Synthase
FN1	Fibronectin 1
GLUT1	Glucose Transporter 1
HIF-1α	Hypoxia-Inducible Factor 1-alpha
HIF-2α	Hypoxia-Inducible Factor 2-alpha
HSL	Hormone-Sensitive Lipase
ICAM-1	Intercellular Adhesion Molecule-1
IL-1β	Interleukin-1 beta
IL-6	Interleukin 6
IL-8	Interleukin 8
JAK/STAT3	Janus kinases/Signal Transducers and Activators of Transcription
LDW	Laser Direct-Write
LPL	Lipoprotein Lipase
MAPK	Mitogen-Activated Protein Kinase
MET	Mesenchymal–Epithelial Transition
MMPs	Metalloproteinases
NF-κB	Nuclear Factor kappa B
obASCs	Obesity-Altered Adipose Stem Cells
PDOs	Patient-derived organoids
PDXs	Patient-derived xenografts
PI3K	Phosphatidylinositol 3-kinase
PPARγ	Peroxisome Proliferator-Activated Receptor Gamma
PLIN1	Perilipin 1
PTGS2	Prostaglandin–Endoperoxide Synthase 2
RAC1	Ras-Related C3 Botulinum Toxin Substrate 1
TAMs	Tumor-Associated Macrophages
TME	Tumor Microenvironment
TNF-α	Tumor necrosis factor alpha
UCP1	Uncoupling Protein 1
VCAM-1	Vascular Cell Adhesion Molecule-1
VEGF	Vascular Endothelial Growth Factor
